# Sex‐biased gene expression in the frontal cortex of common marmosets (*Callithrix jacchus*) and potential behavioral correlates

**DOI:** 10.1002/brb3.1148

**Published:** 2018-10-30

**Authors:** Viviane Brito Nogueira, Danilo Oliveira Imparato, Sandro José de Souza, Maria Bernardete Cordeiro de Sousa

**Affiliations:** ^1^ Health Sciences Graduate Program Federal University of Rio Grande do Norte Natal Brazil; ^2^ Bioinformatics Multidisciplinary Environment Federal University of Rio Grande do Norte Natal Brazil; ^3^ Brain Institute, Bioinformatics Multidisciplinary Environment Federal University of Rio Grande do Norte Natal Brazil; ^4^ Brain Institute and Health Sciences Graduate Program Federal University of Rio Grande do Norte Natal Brazil

**Keywords:** adaptive strategies, database, neuropsychiatric primate model, sexual dimorphism, synaptic plasticity, transcriptomics

## Abstract

**Introduction:**

The common marmoset (*Callithrix jacchus*), a small New World monkey, has been widely used as a biological model in neuroscience to elucidate neural circuits involved in cognition and to understand brain dysfunction in neuropsychiatric disorders. In this regard, the availability of gene expression data derived from next‐generation sequencing (NGS) technologies represents an opportunity for a molecular contextualization. Sexual dimorphism account for differences in diseases prevalence and prognosis. Here, we explore sex differences on frontal cortex of gene expression in common marmoset's adults.

**Methods:**

Gene expression profiles in six different tissues (cerebellum, frontal cortex, liver, heart, and kidney) were analyzed in male and female marmosets. To emphasize the translational value of this species for behavioral studies, we focused on sex‐biased gene expression from the frontal cortex of male and female in common marmosets and compared to humans (*Homo sapiens*).

**Results:**

In this study, we found that frontal cortex genes whose expression is male‐biased are conserved between marmosets and humans and enriched with “house‐keeping” functions. On the other hand, female‐biased genes are more related to neural plasticity functions involved in remodeling of synaptic circuits, stress cascades, and visual behavior. Additionally, we developed and made available an application—the CajaDB—to provide a friendly interface for genomic, expression, and alternative splicing data of marmosets together with a series of functionalities that allow the exploration of these data. CajaDB is available at cajadb.neuro.ufrn.br.

**Conclusion:**

The data point to differences in gene expression of male and female common marmosets in all tissues analyzed. In frontal cortex, female‐biased expression in synaptic plasticity, stress, and visual processing might be linked to biological and behavioral mechanisms of this sex. Due to the limited sample size, the data here analyzed are for exploratory purposes.

## 1 INTRODUCTION

The use of animal models is necessary to advance the understanding of biomedical, evolutionary, and behavioral processes of our species. The common marmoset (*Callithrix jacchus*) is a New World monkey that has been extensively studied in the neuroscience field due to similarities to human brain functioning, circuitry, and behavior (Carlos et al., 2015; Hunt, Carvalho, Pessoa, Mountford, & Davies, 2017; Miller et al., 2016). Similar to humans, marmosets form a sophisticated society based on cooperative breeding (Wobber, Wrangham, & Hare, 2010). They also pair‐bond (Digby & Barreto, 1993; Sousa et al., 2005; Stevenson & Poole, 1976), have rich social signaling systems and cooperatively care for infants (French, 1997; Mota, Franci, & De Sousa, 2006), all features also present in humans. This, together with other marmosets’ characteristics—small size (~300–400 g), easy handling in a lab setting, high reproduction rate, and the possibility of gene editing (Miller et al., 2016)—make them a suitable model for molecular and behavioral studies. Moreover, this species presents a set of pro‐social behaviors that is uncommon among primates, but present in humans (Burkart & van Schaik, 2009).

Brain functioning and behavior rely on both genetic and environmental influences, and there is a substantial recognition that social information can alter brain, behavior, and gene expression. There has been a growing need to understand higher aspects of common marmoset's cognition. Although rodents are widely used in behavioral neuroscience, the most considerable part of the primate prefrontal cortex—part of which is implicated in neuropsychiatric disturbances—has no homolog in other mammals (Wise, 2008). We focused on the frontal cortex since this specific brain region is responsible for many higher behavioral functions that are of vital importance for using marmosets as an experimental model.

In the last decades, the study of biological systems at a molecular level has significantly progressed due to the advent of large‐scale technologies. Omics sciences have great potential to further the understanding of traits in human diseases and represent an opportunity for new biological insights in common marmosets. RNA‐seq provides a more precise measurement of transcripts levels and their isoforms than other transcriptomics methods (Wang, Gerstein, & Snyder, 2009). The differential transcript levels among male and female subjects of the same species are known as sex‐biased gene expression (Grath & Parsch, 2016). Currently, several studies discussed these sex‐biased expression profiles for brain regions in humans and nonhuman primates (Bernard et al., 2012; Fukuoka, Sumida, Yamada, Higuchi, & Nakagaki, 2010; Hawrylycz et al., 2015; Lee et al., 2017; Trabzuni et al., 2013), but only one included common marmosets (Reinius et al., 2008).

Sex differences in gene expression may regulate many biological features including prevalence and/or prognosis of diseases, morphology, neurochemistry, and behavior. Elucidating the molecular basis of such differences is remarkably essential for both basic neurobiology and neuro‐pathophysiology. Despite the importance of this phenomenon, sex differences are still relatively underexplored in neuroscience with a small number of published studies, most lacking a molecular contextualization (Gilks, Abbott, & Morrow, 2014; Trabzuni et al., 2013). As humans and marmosets share cognition and social behavior features (Burkart, Hrdy, & Schaik, 2009; Miller et al., 2016) of which many are relevant for the neuropsychiatric health, the molecular contextualization might provide insights on the biology of sex differences in human neuropsychiatric conditions.

In this study, we analyzed sex‐biased gene expression (RNA‐Seq technology) across tissues and focused on the frontal cortex of common marmosets. To emphasize the translational value of this species we compared marmosets’ data to humans’ expression data in the frontal cortex. Additionally, to facilitate the access and analyses of omics data from marmosets, we developed a user‐friendly application—the CajaDB (https://cajadb.neuro.ufrn.br)—which can be used by the scientific community without solid bioinformatics background.

## 2 MATERIALS AND METHODS

### 2.1 Tissue samples and data source

Common marmoset reference genome (Worley et al., 2014) and transcriptome were downloaded from the UCSC genome browser (version calJac3, RRID: SCR_005780). Public RNA sequencing (RNA‐seq) reads showing high sequence coverage from Cortez et al. (2014) project was downloaded from the Sequence Read Achieve—SRA/NCBI (RRID: SCR_004891). This project included Illumina sequencing data of liver, heart, frontal cortex, cerebellum, kidney, and gonads tissues from male and female marmosets. Human (*Homo sapiens*) genome (version hg19) was downloaded from the UCSC genome browser. RNA‐seq reads of human frontal lobe were downloaded from the SRA/NCBI, data of Brawand et al. (2011) more information on the samples analyzed is available in Supporting Information Table S1.

### 2.2 Data processing

All RNA‐seq reads were mapped using TopHat v2.1. 0 (RRID: SCR_013035) (alignment with Bowtie2—2.2.5, RRID: SCR_005476) (Langmead, Trapnell, Pop, & Salzberg, 2009; Trapnell et al., 2010) to the reference genome assembly. Cufflinks 2.2.1 (RRID: SCR_014597) (Goff, Trapnell, & Kelley, 2012) was used for assembling and to estimate the abundance of transcripts in FPKM (fragments per kilobase of transcript per million mapped reads) values for all genes in the genome. Packages within BioConductor 3.5 (RRID: SCR_006442) (Gentleman et al., 2004) were used for gene expression data analysis.

### 2.3 Sex‐biased gene expression and alternative splicing, and set enrichment analysis

Sex‐biased expression in the frontal cortex of marmoset and human tissues was defined as the normalized difference between expression in males and females: Δ = (*m*−*f*)/(*m* + *f*), where Δ = −1 means female expression only, Δ = 0 means unbiased expression, and Δ = 1 means male expression only (Cheng & Kirkpatrick, 2016) Female‐ and male‐biased genes were defined as the Δ interval of [−1.0−0.5] and [0.5−1.0], respectively (equivalent to *z*‐score 2.0 for genes and 1.5 for isoforms). To identify, Gene Ontology (GO) Consortium (2015, RRID: SCR_002811) categories and Kyoto Encyclopedia of Genes and Genomes (KEGG) (RRID: SCR_012773) pathways enriched for particular subsets of sex‐biased genes, hypergeometric test for overrepresentation was used (*p* < 0.01, enrichGO and enrichKEGG in R). To identify and visualize alternative splicing events (Exon skipping, alt. 5′ border, alt. 3′ border and intron retention), we used the Splicing Express (RRID: SCR_016498) (Kroll, Kim, Ohno‐Machado, & de Souza, 2015).

### 2.4 Statistical analysis

All statistical analyses were performed using R (R Development Core Team, 2009) 3.3 (https://www.R-project.org, RRID: SCR_001905). For enrichment analysis, *p*‐values were adjusted by false‐discovery rate (FDR) (Benjamini & Hochberg, 1995) Enrichment procedures of sex‐biased genes were tested by Monte Carlo simulations (1,000 random sampling sets). During each simulation, a random set was generated with the same size of the investigated set (for male‐ and female‐biased genes). Significance (*p*‐mcarlo) was defined as the number of genes in a given category divided by the number of random sampling (1,000).

### 2.5 Application for interactive visualization of data

The CajaDB (RRID: SCR_016506), a database available in https://cajadb.neuro.ufrn.br, provides a friendly interactive visualization tool for genomic, expression, and alternative splicing data, including tools for enrichment analysis and protein–protein network. More detailed information of this application will be discussed elsewhere.

## 3 RESULTS

### 3.1 Sex‐biased gene expression

**Figure 1:**
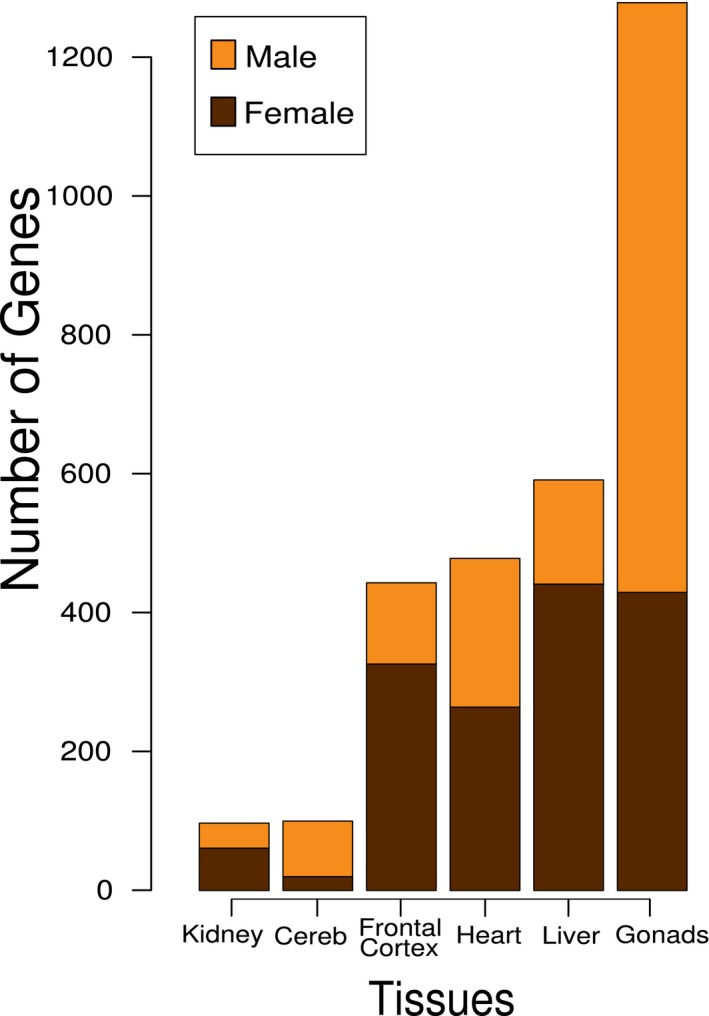
The number of sex‐biased genes in different tissues in common marmosets. About 16.206 are the total of genes described for common marmosets

From the seven tissues analyzed (Figure 1), gonads presented more sex‐biased genes (ovary—429, testis—849), followed by liver (male—150, female—441), heart (male—214, female—264), frontal cortex (male—117, female—326), cerebellum (male—80, female—20), and kidney (male—36, female—61).

**Figure 2:**
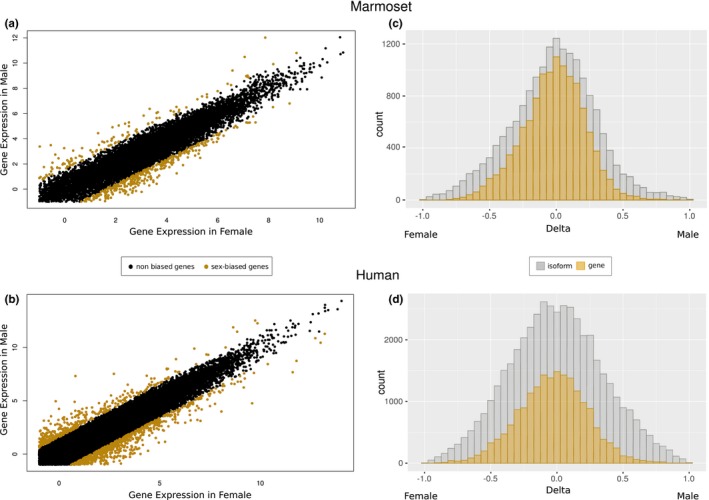
Expressions in the frontal cortex of marmosets and humans. Correlation of all genes by expression in log2 FPKM is presented in marmoset (a) and human (b), where yellow dots are the sex‐biased genes. Distribution of sex‐biased expression, defined as the normalized difference between expression in males and females, by gene‐ and isoform‐levels are shown in (c; marmoset) and (d; human)

The gene expression distribution analysis for frontal cortex of male and female bias showed the same tendency in marmosets and humans (Figure 2a,b), with a slightly higher number of female‐biased genes. As expected, sex‐biased expression was greater at the isoform‐level than at the gene‐level for both species (Figure 2c,d). When sex‐biased expression of humans and marmosets was analyzed, 45.01% were present in both species at isoform‐level (791 out of 1757) but the same pattern was not found at gene‐level analysis.

**Figure 3:**
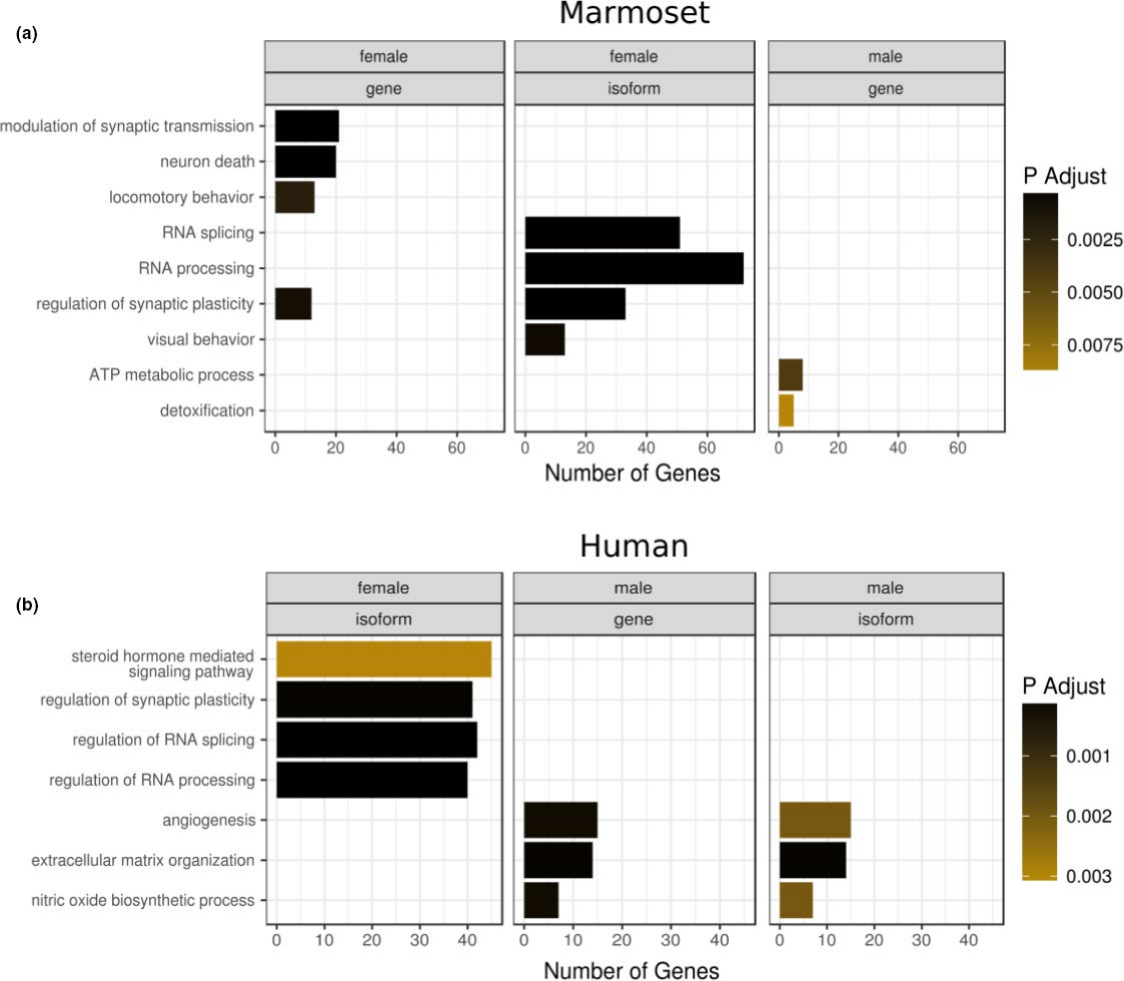
Enrichment analyses for sex‐biased at both isoform‐ and gene‐level. (a) Enrichment analysis (Gene Ontology) for common marmosets. For males, no significant enrichment was found at isoform‐level. (b) Enrichment analysis (Gene Ontology) for humans. For males, no significant enrichment was found at gene‐level

Ontology enrichment analyses were performed to comprehend the biological processes linked to these sex‐biased genes (Figure 3). In general, male‐ and female‐biased genes were enriched for different functional categories: male‐biased genes for more conserved and broadly expressing “house‐keeping” functions whereas female‐biased genes were more related to cognitive functions. A list of all genes and isoforms differentially expressed in females and males are available in Supporting Information Table S2.

We have calculated two *p* values, the first (*p*‐adjust) related the enrichment step and the second to exhibit the robustness of sampling significance over a random set (*p*‐mcarlo). Among male‐biased expression, significant enrichment of genes involved in ATP metabolic processes (*p*‐adjust = 4e−3, *p*‐mcarlo = 1e−4) was found.

Among female‐biased expression, 13 isoforms characterized an enrichment for visual behavior (*p*‐adjust = 5e−4, *p*‐mcarlo = 3e−4) while 33 female‐biased isoforms characterized an enrichment for regulation of synaptic plasticity (*p*‐adjust = 6e−9, *p*‐mcarlo = 6e−4). In humans, 41 female‐biased isoforms characterized an enrichment (*p*‐adjust = 1e−4, *p*‐mcarlo = 7e−4) for regulation of synaptic plasticity with 10 genes (out of the 41) being orthologs of marmosets. Genes into these two categories for marmosets were broadly explored through literature review.

In addition, among female‐biased genes, a significant enrichment of genes related to both RNA splicing (*p*‐adjust = 1e−3, *p*‐mcarlo = 2.9e−3) and RNA processing (*p*‐adjust = 1.49e−9, *p*‐mcarlo = 1e−4) was found.

**Figure 4:**
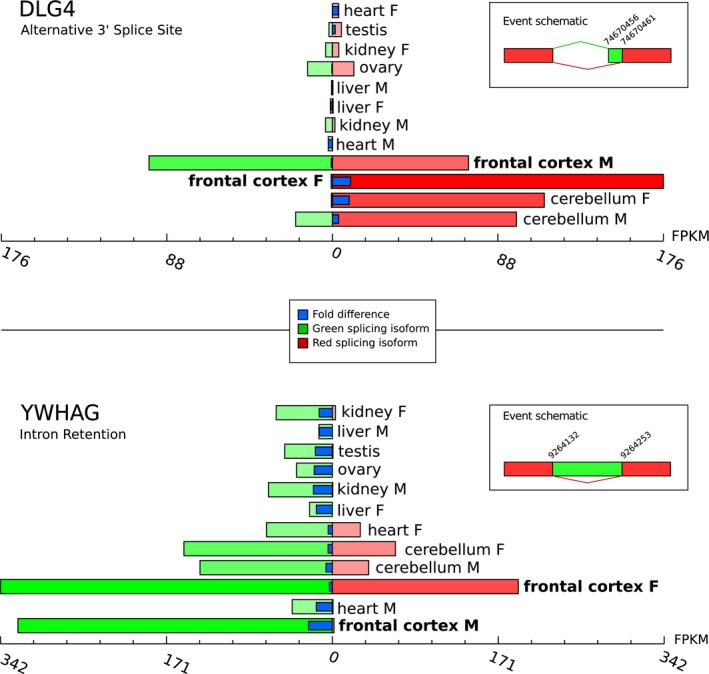
Alternative splicing events in a tissue‐based (frontal cortex, heart, cerebellum, kidney, and liver for male and female, testis and ovary) perspective. Sex‐specific alternative splicing of DLG4 (alternative 3' splice site) and YWHAG (intron retention) can be observed in the frontal cortex. Both genes are involved in regulation of synaptic plasticity

To visualize a tendency in alternative splicing events (ASEs) by sex in our genes of interest (present in the regulation of synaptic plasticity and visual behavior categories), we show ASEs on a tissue‐based perspective (Figure 4). In the DLG4 gene, it was observed a sex‐biased alternative 3' Splice Site event—where the green isoform was expressed in male only, in the frontal cortex. Moreover, in the YWHAG gene, it was observed a sex‐biased intron retention event, where the red isoform was expressed in male only, for frontal cortex.

## 4 DISCUSSION

### 4.1 Sex differences in molecular context

Tissue‐based studies commonly show that sex‐biased expression tends to be higher in the gonads compared to other tissues (Albritton et al., 2014; Mayne et al., 2016). In our data, we found the same tendency. Nonetheless, sex‐biased expression has been described for other species in kidney (Kwekel, Desai, Moland, Vijay, & Fuscoe, 2013), cerebellum (Ziats & Rennert, 2014), frontal cortex (Xu et al., 2014), heart (Isensee et al., 2008), and liver (Zhang et al., 2011). These biases in expression are important regarding sex differences in disease susceptibility related to these tissues (Perucca, Bouby, Valeix, & Bankir, 2007; Regitz‐Zagrosek & Kararigas, 2017; Werling & Geschwind, 2013).

We focused on sex‐biased expression in the frontal cortex of marmosets and compared to humans. All the comments from now on are on behalf of this brain tissue. Gene expression is determining to promote key male and female traits, and it may be conserved during evolution (Carlos et al., 2015; Reinius et al., 2008). We found, for both species analyzed, that sex‐biased expression was greater at the isoform‐level than at the gene‐level, as expected (Djebali et al., 2012). Additionally, 45.01% of sex‐biased genes at isoform‐level were present for human and marmoset species.

Sex hormones have been shown to influence on neurogenesis, cell differentiation, apoptosis, axon guidance, and synaptogenesis processes (Jazin & Cahill, 2010). In our enrichment analysis for frontal cortex, sex‐biased genes fell into categories related to all these processes. We focused the discussion on the genes that fell into the categories: regulation of synaptic plasticity, visual behavior, and RNA splicing.

### 4.2 Synaptic plasticity

Synaptic plasticity (SP) is defined as the process of strengthening or weakening synapses related to development or learning. SP pathways have been widely discussed in the sex‐bias context (Bourgeron, 2015; Dachtler & Fox, 2017; Duman, Aghajanian, Sanacora, & Krystal, 2016). In our marmoset's data, the female‐biased genes NLGN1, RASGRF1, PRKCZ, SRF, IQSEC2, JPH4, UNC13A, YWHAG, KCNB1, STXBP1, BRAF, and SNAP25 are associated with general functional synaptic plasticity. Some other genes are linked to morphological (growth or apoptotic) mechanisms: TNR, CNTN4, STAU1, PPP1R9A, CAMK2B, and SNCA. Sex differences do not simply reflect differences in gonadal hormones, but also reveal distinctions in synaptic signaling mechanisms (Mizuno & Giese, 2010). One of the factors that are involved with sex‐biased differences is related to CaMKK and estrogen receptor pathways which present sexual dimorphism with implications for SP in the cerebral cortex (Dachtler & Fox, 2017). Additionally, glutamate (L‐Glu) is the main and most abundant excitatory neurotransmitter in the central nervous system of mammals, playing a crucial role in the mechanisms underlying SP. These mechanisms depend on stimulation of several glutamate receptors. In marmosets, some female‐biased genes are specifically related to the glutamatergic system: CPEB3, DLG4, SHISA9, SHISA7, FMR1, ABHD6, and NPTN. These sex differences in glutamatergic pathways are supported by findings reviewed by Dachtler and Fox (2017).

In the category of regulation of SP, female‐biased genes were found in marmosets as well as in humans with 10 genes being orthologs. In this regard, the similarity of these results points for homologous neural mechanisms across primate species, as discussed previously (Platt, Seyfarth, & Cheney, 2016; Wilson, Marslen‐Wilson, & Petkov, 2017). These well‐described sex differences in the plasticity functionalities are in accordance with our exploratory data of marmosets.

### 4.3 Gene expression on stress and social behavior

Changes in the social environment require changes in behavior. At the molecular level, they rely on the regulation of gene expressions by signaling pathways. This molecular response to perceived social information is known as social plasticity. Differences in expression can emulate sex‐biased gene regulatory structures and have been reported having functional importance on behavior (Burmeister, Jarvis, & Fernald, 2005; Pointer, Harrison, Wright, & Mank, 2013; Trabzuni et al., 2013). Males and females’ marmosets present different strategies in social behavior. In general, marmosets are considered cooperative breeders which are characterized as predominantly monogamous (Arruda et al., 2005; Sousa et al., 2005). This might be involved with differences in their reproductive strategy, where females reproductive strategies are based on competition whereas males are based on cooperation (Yamamoto, et al., 2010). From the female‐biased genes of marmosets on our analysis, NTRK2, CREB1, and CRTC1 are possibly associated with CREB1‐BDNF‐NTRK2 pathway which plays a significant role in brain adaptation to stress (Juhasz et al., 2011), suggesting a higher demand for this signaling cascade in females. Additionally, sex differences in stress are widely described in many mammals (Palanza & Parmigiani, 2017). In common marmosets, sex‐different stress reactivity (cortisol levels) in the context of reproductive pairs separation was demonstrated by Sousa, Leão and Silva (2002).

### 4.4 Visual processing

The genes in the visual behavior category (PIAS1, APP, NDRG4, KMT2A, NLGN3, HMGCR, HIF1A, CDK5, ATP1A3, MECP2, and SLC24A2) may be associated with different visual processing between sexes. The prefrontal cortex (PFC) is primarily an integrative cortex, where sensory and other inputs determine and guide commands and decisions. Thus, differential gene expressions in the frontal cortex may be related to female executive and mothering functions and/or intrasexual competition. It is possible that marmoset females show a higher sustained attentional control, which is necessary for infant care, particularly at an earlier infant age when additional help is critical for her reproductive success (Arruda et al., 2005). Therefore, differences in visual processing of males and females might account for sex differences and a higher demand for visual processing in females.

### 4.5 Alternative splicing

RNA splicing and processing categories were significantly enriched for female‐biased genes in humans and nonhuman primates. The concepts of (a) alternative splicing is one of the main mechanisms controlling the large variability of mRNA and protein isoforms, and (b) sex‐bias in alternative splicing is a relevant biological mechanism underlying sex differences (McIntyre et al., 2006; Stolc et al., 2004). Our analysis shows a difference in the tendency of alternative splicing events between sexes, which is consistently described in humans and nonhuman primates (Blekhman, Marioni, Zumbo, Stephens, & Gilad, 2010). Alternative splicing data for common marmosets can be further explored in our web application.

### 4.6 CajaDB

CajaDB, a molecular database of marmosets, provides an intuitive interface to visualize and explore genomic, transcriptomic, and alternative splicing data. Our application not only allows the user to navigate the data but also supports biological analyses such as functional (ontology) enrichment analysis and protein–protein network. Hopefully, these centralized resources will provide numerous benefits to researchers in addressing scientific questions. More detailed information of this application will be provided elsewhere.

### 4.7 Research limitations

We would like to make clear that this study is limited by the sample size, and we present it as an exploratory analysis. We found that genes whose expression is male‐biased are conserved between marmosets and humans and enriched with house‐keeping functions whereas the female‐biased genes are more related to neural plasticity (remodeling of synaptic circuits, stress cascades, and visual behavior). This observation, impressively, seems to be highly associated with differences in social behavioral strategies between sexes. This is significant because common marmosets are a very important experimental model in neuroscience, and these differences might account for the investigation of neuropsychiatric disorders. We have not applied the gold standard method for differential gene expression analysis due to the limited data. We used then the delta metrics aiming to present a preliminary analysis of sex‐bias gene expression in common marmosets. Unfortunately, at this moment, there is no possibility of expanding our sample size because our work uses public transcriptomic data, and there is no additional data available for common marmosets. Nonetheless, the concordance of the transcriptomic analysis with the great mass of work at the behavioral level made us believe that this work brings a contribution to the behavioral studies at the molecular level.

## 5 CONCLUSION

Knowledge of the brain circuitry that drives social interactions is limited, in part due to the technical limitations of measuring brain activity in humans. Animal models have been and will continue to be useful to study many aspects of behavior, particularly to decipher the molecular basis of human social behavior. Unfortunately, to date, few behavioral paradigms use experimental animal models to study the neural basis of social behavior. Findings of our study emphasize the translational value of common marmosets. Female‐biased genes in frontal cortex were enriched toward cognitive functions while male‐biased genes were associated with “house‐keeping” functions. This relationship was also present when we analyzed expression data from humans. Female‐biased expression in the frontal cortex of marmosets might be involved with a more resourceful stress cascade to lead with social competition present in this sex as well as a higher sustained attentional control on their visual goal‐directed behavior.

## 6 FUTURE DIRECTIONS

Here, we presented a data exploration analysis. To validate this data, we suggest follow‐up experiments (with a higher number of testing individuals) designed to test, for instance, the sex differences in the expression of genes involved in synaptic plasticity, stress behavior context, and visual goal‐directed behavior. In marmosets, social groups are composed of the breeding pair and other mature and immature individuals (Abbott, Saltzman, Schultz‐Darken, & Tannenbaum, 1998; Gilks et al., 2014). Female × female relationship demands positive and aggressive interactions into the social group to reach and to maintain the dominance. It differs from male x male social dynamic, in which lower aggressive display is observed (Yamamoto et al., 2010). Sex‐biased gene expression in the context of dominance was analyzed in birds: when subordinate males were compared to the dominants, the overall expression patterns were concordant with their phenotypic status (Pointer et al., 2013). We envision that gene expression patterns would be a good strategy to investigate social hierarchy in marmosets.

## CONFLICT OF INTEREST

None declared.
